# Rabies in a Dog Imported from Egypt — Kansas, 2019

**DOI:** 10.15585/mmwr.mm6938a5

**Published:** 2020-09-25

**Authors:** Chelsea Raybern, Allison Zaldivar, Sheri Tubach, Farah S. Ahmed, Susan Moore, Caitlin Kintner, Ryan M. Wallace, Anna M. Mandra, Kendra Stauffer, Rene Edgar Condori, Ingrid Garrison

**Affiliations:** ^1^Kansas Department of Health and Environment; ^2^Kansas State University Rabies Laboratory, Manhattan, Kansas; ^3^Johnson County Department of Health and Environment, Olathe, Kansas; ^4^Division of High-Consequence Pathogens and Pathology, National Center for Emerging and Zoonotic Infectious Diseases, CDC; ^5^Division of Global Migration and Quarantine, National Center for Emerging and Zoonotic Infectious Diseases, CDC.

Although canine rabies virus variant (CRVV) was successfully eliminated from the United States after approximately 6 decades of vaccination campaigns, licensing requirements, and stray animal control, dogs remain the principal source of human rabies infections worldwide. A rabies vaccination certificate is required for dogs entering the United States from approximately 100 countries with endemic CRVV, including Egypt (*1*). On February 25, 2019, rabies was diagnosed in a dog imported from Egypt, representing the third canine rabies case imported from Egypt in 4 years (*2*,*3*). This dog and 25 others were imported by a pet rescue organization in the Kansas City metropolitan area on January 29. Upon entry into the United States, all 26 dogs had certificates of veterinary inspection, rabies vaccination certificates, and documentation of serologic conversion from a government-affiliated rabies laboratory in Egypt. CDC confirmed that the dog was infected with a CRVV that circulates in Egypt, underscoring the continued risk for CRVV reintroduction and concern regarding the legitimacy of vaccine documentation of dogs imported from countries considered at high risk for CRVV. Vaccination documentation of dogs imported from these countries should be critically evaluated before entry into the United States is permitted, and public health should be consulted upon suspicion of questionable documents.

## Investigation and Findings

On January 28, 2019, 26 dogs arrived at the Pearson International Airport in Toronto, Canada, from Cairo, Egypt. The dogs were driven from Canada to the Kansas City metropolitan area through Port Huron, Michigan. The dogs’ documentation was reviewed by Canadian authorities, the United States Border Patrol, and the Kansas Department of Agriculture and met entry requirements. The dogs were immediately adopted or fostered by persons in Kansas and Missouri upon arrival.

On February 20, a fostered 2-year-old dog from this cohort (dog A) developed polydipsia, polyphagia, and diarrhea. The next evening, it began vomiting, ingested a blanket, and developed ataxia, hypersalivation, and abnormal vocalization. After transport to veterinary hospital A on February 21, dog A displayed abnormal aggression and bit a technician. The dog was transferred to veterinary hospital B on February 22 and exhibited bilateral protruding third eyelids and on February 23 was observed biting at the air as if trying to catch a fly (i.e., fly-biting behavior), both of which are considered neurologic abnormalities consistent with rabies virus infection. The dog continued to decline as it became laterally recumbent and developed increased aggression. Veterinary staff members at hospital B suspected rabies, and the dog was humanely euthanized and submitted for rabies testing on February 24.

On February 25, the Kansas State University Rabies Laboratory (KSU-RL) confirmed rabies infection by direct fluorescent antibody test. On March 1, CDC identified the cosmopolitan canine rabies virus lineage by sequencing the complete nucleoprotein (N) gene. The sequence was nearly identical to virus from a rabid dog imported into Connecticut from Egypt in 2017, with six nucleotides substituted (99.5% identical) across the entire N gene.

## Public Health Response

After KSU-RL confirmed rabies, the Kansas Department of Health and Environment (KDHE), Johnson County Department of Health and Environment, Missouri Department of Health and Senior Services, Kansas Department of Agriculture, Missouri Department of Agriculture, United States Department of Agriculture, and CDC initiated an investigation to implement prevention and control measures.

KDHE, Missouri Department of Health and Human Services, and Johnson County Department of Health and Environment interviewed dog A’s caretakers, pet rescue director, and staff members of veterinary hospitals A and B to assess potential human and animal exposures. Overall, 44 persons elected to receive rabies postexposure prophylaxis (PEP), 38 (86%) of whom were veterinary staff members who initiated PEP before assessment by public health. After assessments were conducted, the departments of health recommended that only 19 of those persons receive PEP, including 13 veterinary staff members, five pet rescue employees, and one household contact. Eighteen (95%) of the 19 were nonbite exposures.

Dog A had been fostered with 12 other dogs and two cats from the United States. Two of the 12 dogs were not immunized against rabies (one was pregnant at the time it was acquired by the pet rescue so did not receive rabies vaccination and the other was not vaccinated for unknown reasons). These two dogs were placed in a 6-month quarantine at the pet rescue. The other 10 dogs were administered rabies booster vaccinations and observed for 45 days. The two cats were never exposed to dog A.

Serologic assays (rapid fluorescent focus inhibition test [RFFIT] and enzyme-linked immunosorbent assay [ELISA]) were performed by KSU-RL on serum drawn from dog A before euthanasia to determine if it had evidence of past vaccination. RFFIT, which measures neutralizing function of antibodies, was positive (0.8 IU/mL). ELISA, which measures binding immunoglobulin (Ig) G antibodies to viral antigens, was negative (<0.125 EU/mL), indicating that the neutralizing antibody detected with RFFIT was IgM. Vaccination was reported to have occurred >2 months earlier; since IgM response occurs shortly after antigen exposure, and IgG response is detectable after Ig class-switching and is long-lived, these results indicate dog A had no history of vaccination but was in the early stage of development of rabies infection at the time it was euthanized.

Because of uncertainty about the validity of documentation or efficacy of rabies vaccine administered in Egypt, KDHE required the remaining 25 dogs to be quarantined or euthanized. All 25 dogs were returned to the pet rescue, which was approved by the Kansas Department of Agriculture’s Animal Facilities Inspection Program, for quarantine by March 1. Length of quarantine was determined through prospective serologic monitoring, which is recognized by the National Association of State Public Health Veterinarians as a testing method to evaluate whether a healthy dog or cat without valid rabies vaccine documentation has been previously vaccinated (*4*). Prospective serologic monitoring utilizes RFFIT on paired serum specimens collected on days 0 and 5–7. Rabies vaccine is administered after collection of the first specimen. If the first titer is ≥0.5 IU/mL or a statistically significant rise in titer (1.8-fold increase) occurs between collection of the first and second specimen, and the second titer is ≥0.5 IU/mL, then the animal is considered to have been previously vaccinated.

All 26 imported dogs had documentation of recent receipt of rabies vaccine from three different manufacturers (Table). These manufacturers confirmed that all vaccine products listed on the certificates were valid products based on lot numbers. KSU-RL performed prospective serologic monitoring. Seven dogs (B, D, G, H, J, N, and Z) had serologic evidence of previous vaccination and were quarantined for 4 months. The remaining 18 dogs had no evidence of previous vaccination and required a 6-month quarantine. Quarantine release dates were calculated from the dogs’ arrival in North America (January 28); all dogs survived and were released on May 29 or July 29, 2019. The other dogs considered to have been exposed to the rabid dog at the foster home also survived their quarantine/observation periods.

**TABLE:** Vaccination dates and antibody titer results for dogs imported into the United States from Egypt (N = 26) — Kansas, 2019

Dog	Vaccine	Age, yrs	Egypt			Kansas State University Rabies Laboratory				Evidence of previous vaccination
			Date of rabies vaccine	Date of titer collection	Titer result (IU/mL)	First titer date of collection	First titer result (IU/mL)	Second titer date of collection	Second titer result (IU/mL)	
A*	V1A	2.2	Dec 1, 2018	Jan 15, 2019	0.7	Feb 21, 2019	0.8	N/A	N/A	No
B	V1A	2.2	Nov 1, 2018	Jan 8, 2019	0.8	Mar 5, 2019	0.7	Mar 10, 2019	3.8	Yes
C	V1A	2.2	Nov 1, 2018	Jan 8, 2019	0.9	Mar 5, 2019	<0.1	Mar 10, 2019	<0.1	No
D	V1A	0.9	Nov 1, 2018	Jan 8, 2019	0.9	Mar 5, 2019	0.1	Mar 10, 2019	3.8	Yes
E	V1A	2.2	Dec 1, 2018	Jan 15, 2019	0.8	Mar 5, 2019	<0.1	Mar 10, 2019	0.3	No
F	V1A	2.2	Dec 1, 2018	Jan 15, 2019	0.9	Mar 5, 2019	<0.1	Mar 10, 2019	0.1	No
G	V1A	1.2	Dec 1, 2018	Jan 15, 2019	0.7	Mar 5, 2019	0.2	Mar 10, 2019	0.6	Yes
H	V1A	4.2	Dec 1, 2018	Jan 15, 2019	0.8	Mar 5, 2019	3.1	Mar 10, 2019	4.8	Yes
I	V1B	2.1	Nov 1, 2018	Jan 8, 2019	0.9	Mar 5, 2019	<0.1	Mar 10, 2019	0.1	No
J	V1B	8.0	Nov 1, 2018	Jan 8, 2019	1.0	Mar 5, 2019	0.6	Mar 10, 2019	0.7	Yes
K	V1B	1.7	Nov 1, 2018	Jan 8, 2019	0.9	Mar 5, 2019	<0.1	Mar 10, 2019	<0.1	No
L	V1B	3.8	Nov 1, 2018	Jan 8, 2019	1.0	Mar 5, 2019	<0.1	Mar 10, 2019	0.1	No
M	V1B	2.3	Dec 1, 2018	Jan 15, 2019	0.9	Mar 5, 2019	<0.1	Mar 10, 2019	0.1	No
N	V1B	1.9	Dec 1, 2018	Jan 15, 2019	0.7	Mar 5, 2019	2.8	Mar 10, 2019	3.3	Yes
O	V1C	3.5	Dec 1, 2018	Jan 16, 2019	0.9	Mar 5, 2019	<0.1	Mar 10, 2019	<0.1	No
P	V1C	1.1	Dec 1, 2018	Jan 17, 2019	0.8	Mar 5, 2019	<0.1	Mar 10, 2019	<0.1	No
Q	V1C	2.6	Dec 1, 2018	Jan 18, 2019	1.0	Mar 5, 2019	≤0.1	Mar 10, 2019	<0.1	No
R	V1C	1.1	Dec 1, 2018	Jan 19, 2019	0.7	Mar 5, 2019	<0.1	Mar 10, 2019	<0.1	No
S	V1C	1.3	Dec 1, 2018	Jan 20, 2019	0.9	Mar 5, 2019	<0.1	Mar 10, 2019	<0.1	No
T	V1C	4.2	Dec 1, 2018	Jan 21, 2019	0.8	Mar 5, 2019	0.1	Mar 10, 2019	0.1	No
U	V2	2.2	Nov 1, 2018	Jan 8, 2019	0.9	Mar 5, 2019	<0.1	Mar 10, 2019	<0.1	No
V	V2	0.7	Nov 1, 2018	Jan 8, 2019	0.8	Mar 5, 2019	<0.1	Mar 10, 2019	<0.1	No
W	V2	2.7	Nov 1, 2018	Jan 8, 2019	1.0	Mar 5, 2019	≤0.1	Mar 10, 2019	<0.1	No
X	V2	2.7	Nov 1, 2018	Jan 8, 2019	1.0	Mar 5, 2019	<0.1	Mar 10, 2019	<0.1	No
Y	V2	1.7	Nov 1, 2018	Jan 8, 2019	0.9	Mar 5, 2019	<0.1	Mar 10, 2019	<0.1	No
Z	V3	1.8	Nov 1, 2018	Jan 8, 2019	0.9	Mar 5, 2019	3.1	Mar 10, 2019	3.1	Yes

**Abbreviation:** N/A = not applicable.

* Dog had a positive test for rabies.

## Discussion

CDC estimates that 1.06 million dogs enter the United States each year; 107,100 from areas considered to be high risk for endemic CRVV (*5*). Countries are considered high risk for exporting a dog infected with CRVV when the virus is enzootic anywhere within the country and their rabies surveillance and dog vaccination programs do not meet the standards developed by the World Organisation for Animal Health (*6*).

Each imported case of CRVV represents a risk of reintroduction of the virus into the United States canine population and exhausts public health resources. Each response to an imported dog with CRVV is estimated to consume 800 hours in resources and cost nearly $214,000 in personnel time and PEP (*7*). During this investigation, an average of $9,290* was spent per person for PEP, excluding administration and exam charges, totaling $176,510 for 19 persons who were recommended to receive PEP or $408,760 for all 44 persons who received PEP.

Federal regulation requires that dogs imported into the United States from CRVV high-risk countries have a valid rabies vaccination certificate documenting receipt of vaccine at least 28 days before travel (*1*). Kansas regulation requires dogs to have a certificate of veterinary inspection issued 30 days before movement and proof of rabies vaccination in animals aged >3 months (*8*). These documents were examined at the Canada–United States and Kansas borders for all dogs, and vaccine lot numbers were verified with manufacturers listed on the rabies certificates. Results from serologic testing performed in Egypt suggested that all dogs had mounted sufficient immune responses to a previous vaccination; however, prospective serologic monitoring results indicated that only seven of 25 dogs had evidence that they had responded to a prior rabies vaccination. Serology results were unable to be verified by the Egyptian laboratory because of invalid contact information.

This is the third importation of a rabid dog from Egypt in 4 years; the other two dogs were imported into Connecticut (*2*) and Virginia (*3*), and all three were rescue dogs with rabies vaccination certificates upon U.S. entry. It is not known if the insufficiency of dog A’s rabies vaccination was a result of inadequate vaccine potency related to improper storage and handling, vaccination failure, or fraudulent documentation. Prospective serologic monitoring results of the remaining dogs confirm a systemic failure representing either the inability to appropriately deliver rabies vaccine or forgery of importation documents. The rabid dog in Virginia was determined to have an intentionally falsified rabies vaccination certificate (*3*). To prevent the importation of rabid dogs into the United States, CDC suspended dog importations from Egypt on May 10, 2019 (*7*). Given the frequency and high cost associated with these investigations, this event highlights the importance of thorough review of vaccination and serology documents for dogs imported from countries lacking robust veterinary safeguards and consultation with public health officials upon suspicion of fraudulent or inconsistent records.

**Summary**

**What is already known about this topic?**

Canine rabies virus variant has been eliminated from the United States; however, importation of dogs from high-risk countries risks reintroduction. Investigation of an imported rabid dog from Egypt in Virginia in 2015 revealed the dog had a falsified rabies vaccination certificate.

**What is added by this report?**

Among 26 dogs imported into Kansas from Egypt in 2019, one had rabies; rabies vaccination certificates and certificates of veterinary inspection accompanied all dogs. U.S. serologic testing confirmed that most dogs had never received rabies vaccine.

**What are the implications for public health practice?**

The reason for inadequacy of the rabid dog’s vaccination in unknown. Vaccination documentation of dogs imported from high-risk countries should be critically evaluated before dogs enter the United States.

## Footnotes

* Average rabies PEP cost per person was calculated using dose and price information provided by three health care facilities in Kansas for 17 persons who received PEP in response to this rabid dog investigation. Price only includes cost of the biologics themselves and does not include any additional fees (e.g., administration fee or emergency department fee).
